# Single Walled BiI_3_ Nanotubes Encapsulated within Carbon Nanotubes

**DOI:** 10.1038/s41598-018-28446-2

**Published:** 2018-07-04

**Authors:** Anumol Erumpukuthickal Ashokkumar, Andrey N. Enyashin, Francis Leonard Deepak

**Affiliations:** 1 Nanostructured Materials Group, Department of Advanced Electron Microscopy, Imaging and Spectroscopy, International Iberian Nanotechnology Laboratory (INL), Avenida Mestre Jose Veiga, Braga, 4715-330 Portugal; 2 Institute of Natural Sciences and Mathematics, Ural Federal University, Turgeneva Str., 4, 620083 Ekaterinburg, Russian Federation; 3 Institute of Solid State Chemistry, Ural Branch of Russian Academy of Sciences, Pervomayskaya Str., 91, 620990 Ekaterinburg, Russian Federation

## Abstract

Inorganic nanotubes are morphological counterparts of carbon nanotubes (CNTs). Yet, only graphene-like BN layer has been readily organized into single walled nanotubes so far. In this study, we present a simple route to obtain inorganic single walled nanotubes - a novel ultrathin morphology for bismuth iodide (BiI_3_), embedded within CNTs. The synthesis involves the capillary filling of BiI_3_ into CNT, which acts as a nanotemplate, by annealing the BiI_3_-CNT mixture above the melting point of BiI_3_. Aberration corrected scanning/transmission electron microscopy is used in characterizing the novel morphology of BiI_3_. A critical diameter which enables the formation of BiI_3_ nanotubes, against BiI_3_ nanorods is identified. The relative stability of these phases is investigated with the density functional theory calculations. Remarkably, the calculations reveal that the single walled BiI_3_ nanotubes are semiconductors with a direct band gap, which remain stable even without the host CNTs.

## Introduction

Single walled nanotubes (SWNTs) of inorganic compounds akin to those of carbon are an outstanding set of nanomaterials that have attracted great attention for a variety of properties and applications^1–3^. Although the multi-walled counterparts are readily synthesized in the case of layered compounds including those of MoS_2_, WS_2_, etc. the synthesis of the SWNTs in the case of inorganic compounds poses a great challenge and a hurdle for investigating novel phenomena, properties and applications^4,5^. The formation thermodynamics of inorganic SWNTs is associated only to their high strain energies, not involving naturally any interlayer interaction. Hence, narrow SWNTs of inorganic compounds have been predicted to be less stable than their multi-walled counterparts^6,7^. This might explain the great difficulty, for example, in the synthesis of SWNTs of MoS_2_ and WS_2_, etc. However this challenge has been overcome recently with the synthesis of SWNTs of PbI_2_ with the aid of CNTs which served as ideal templates for their formation^3^. Single layered metal halides of CeI_3_, CeCl_3_, TbCl_3_ and ZnI_2_ have also been synthesized by this approach^8^. The initial studies on opening up of carbon nanotubes and their encapsulation with metals and oxides were performed by Ajayan and Iijima as well as by Green and coworkers^9–11^. Dujardin *et al*. observed that the surface tension of materials play an important role in capillarity, with a low surface tension leading to wetting of CNT surface and capillary filling^12^. Subsequently, the hollow interior of CNTs was shown to provide a unique environment for the filling of molecules, compounds and meta-stable phases and serves as an ideal template for the growth of nanostructures with novel properties^13–16^. This unique feature of CNT has been exploited in the present study wherein the formation of BiI_3_ SWNTs has been achieved starting from MWCNTs (multi-walled CNTs) as templates.

**Figure 1:**
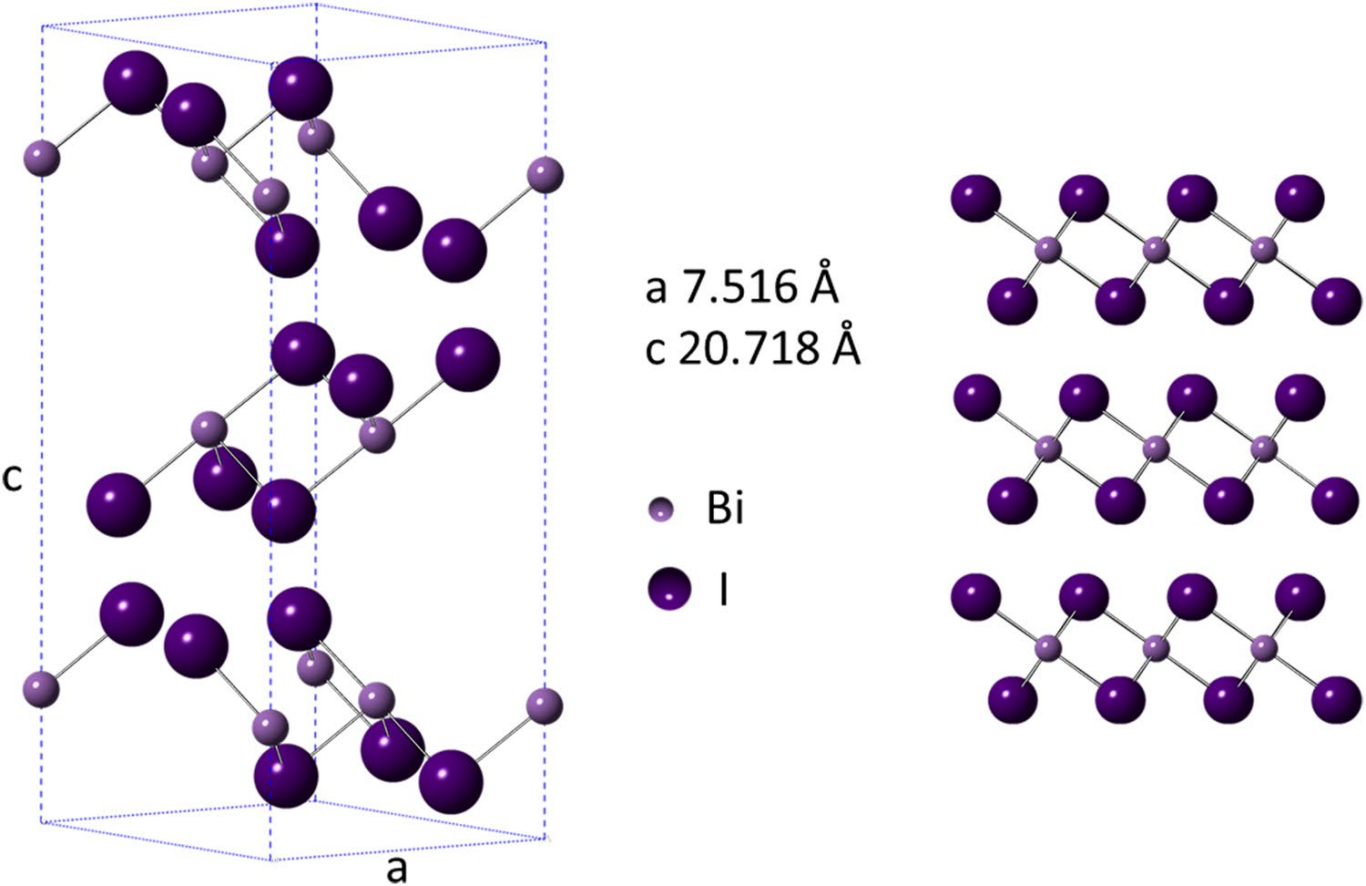
Structural model of rhombohedral BiI_3_ showing the unit cell (left) and the crystal along (100) view direction showing the layered structure (right).

BiI_3_ is a layered semiconducting material having a wide band gap reported to be 1.67 eV. A single layer BiI_3_ is a sandwich-like structure with Bi^3+^ ions establishing six-fold coordination with I^−^ ions. The BiI_3_ layers are held together through weak van der Waals forces, allowing BiI_3_ crystals to be cleaved readily along the [00 l] direction^17–19^. A schematic structural model of BiI_3_ unit cell along with the unit cell parameters is shown in Fig. 1. The layered nature of BiI_3_ is also shown. BiI_3_, having high effective atomic number and high absorption coefficient, is an important material in radiation detection. Thus it has potential applications in room-temperature γ-ray detectors and X-ray digital imaging sensors^20,21^. Recently, appreciable interest has been directed to the optical properties of BiI_3_ because of its strong intrinsic optical anisotropy and the potential for “defect-tolerant” charge transport properties, making it a suitable candidate as thin-film photovoltaic absorber as well as acceptors in inorganic–organic solar cells^22^.

Multi-walled core–shell BiI_3_@WS_2_ inorganic nanotubes (INT) were synthesized previously by employing the molten salt technique which basically involved the wetting and capillary filling of liquid BiI_3_ into the INT template^23^. Nanotubes of mono elemental Bi have been reported earlier^24–26^. One-dimensional nanowires of Bi hosted within SWNTs have also been previously synthesized by heating solid bismuth nanoparticles with SWCNTs^27^. In the present study, the formation of single walled BiI_3_ nanotubes encapsulated within CNT is reported for the first time; achieved by annealing the bulk powder of BiI_3_ along with the host CNT at temperatures slightly above the melting point of BiI_3_. Subsequently in-depth characterization of the morphology, structure and phase was carried out by aberration-corrected high-resolution transmission electron microscopy (AC-HRTEM) and high angle annular dark field scanning transmission electron microscopy (HAADF-STEM) imaging in combination with energy dispersive X-ray spectroscopy (EDX) and electron energy loss spectroscopy (EELS). A threshold diameter for the formation of the nanotubes in comparison to their counterparts - nanorods - was clearly established. In order to understand the formation peculiarities of these novel BiI_3_ phases, density functional theory (DFT) calculations were carried out, to establish their relative stability and electronic properties.

## Results and Discussions

**Figure 2:**
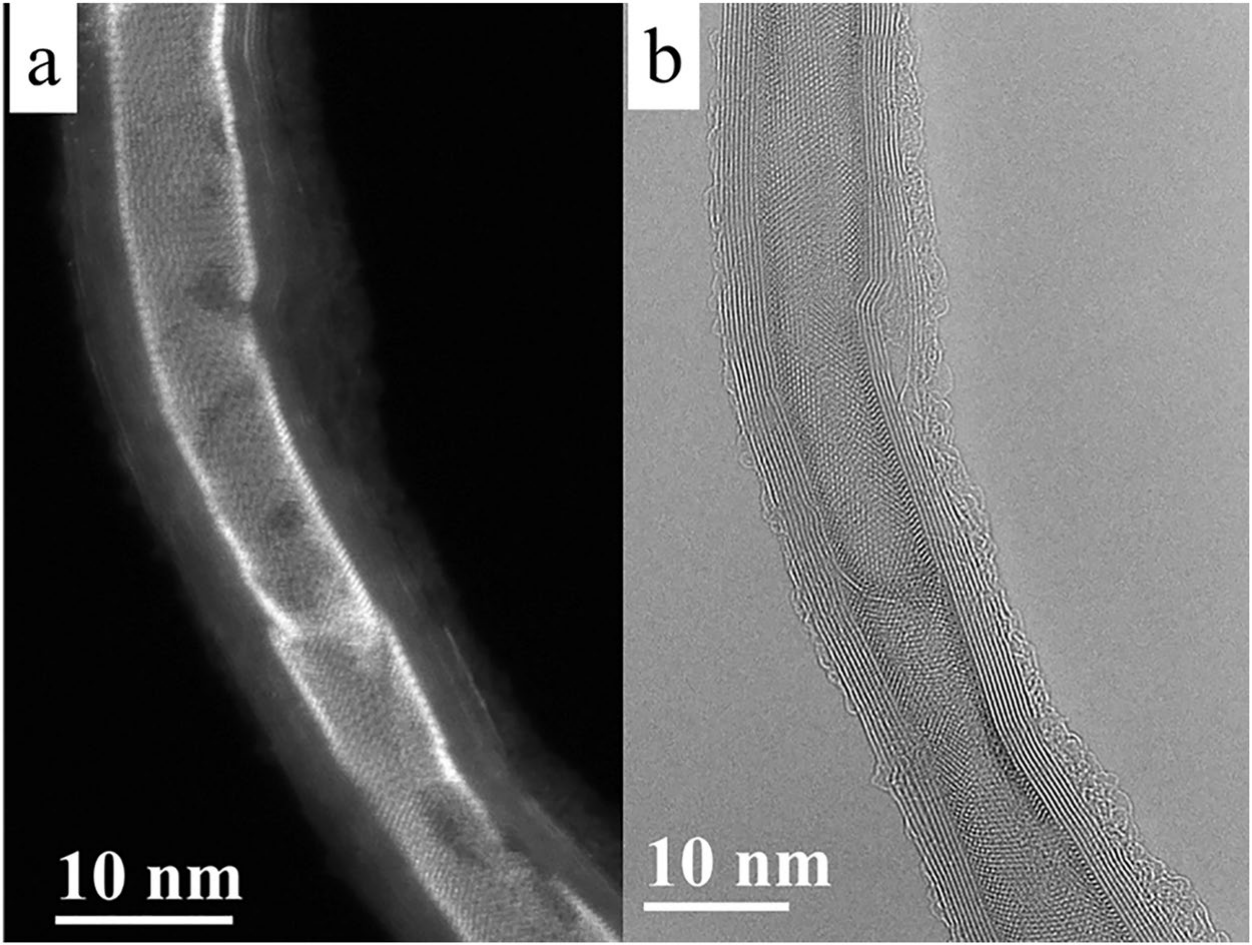
BiI_3_ NT@CNT. (**a**) HAADF-STEM image and (**b**) AC-HRTEM image showing the encapsulation of BiI_3_ as a single walled nanotube within CNT.

Figure 2 shows the typical morphology of BiI_3_ NT@CNT obtained after capillary filling method. In the HAADF-STEM image, BiI_3_ is seen as the brighter region within the darker CNT region (with 8 walls) due to the Z dependence of the image contrast^28^. The BiI_3_ nanotube walls appear particularly brighter in this image. The contrast due to the single walled nature of the BiI_3_ is seen in the TEM image shown in Fig. 2b as well. The BiI_3_ SWNT formed is single crystalline in nature, equivalent to a 2D sheet of BiI_3_ wrapped into a cylinder as shown in Supplementary Fig. S1. The FFT of a region in Supplementary Fig. S1 is in agreement with the simulated diffraction pattern of the tube viewed along the [001] zone axis of the rhombohedral BiI_3_ crystal (PDF Number: 00-007-0269), shown in Supplementary Fig. S1d.

**Figure 3:**
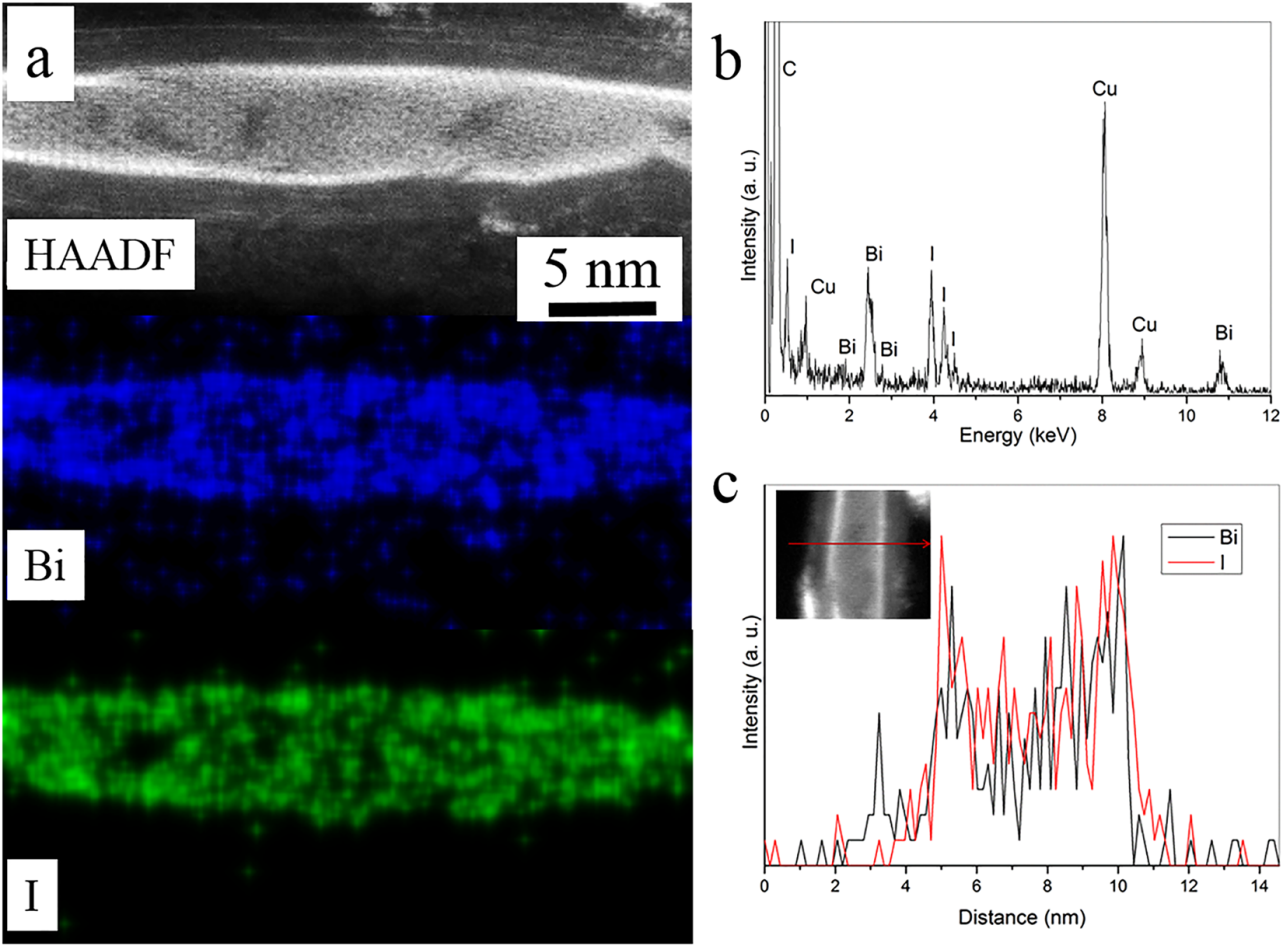
BiI_3_ NT@CNT. (**a**) HAADF-STEM image and EDX elemental maps showing Bi and I. (**b**) EDX spectrum showing C, Bi, I peaks. (**c**) EDX line profile for Bi and I along the line across the BiI_3_ NT@CNT shown in inset, confirming the tube morphology of BiI_3_ within CNT.

STEM-EDX elemental mapping of the BiI_3_ NT@CNT confirms the presence of Bi and I (Fig. 3a) and the quantification of the EDX spectrum reveals the elemental Bi:I ratio as 1:3, corresponding to BiI_3_ (Fig. 3b, Supplementary Table S3). The EDX line profiles in Fig. 3c indicate a single walled tube with the highest intensity of Bi and I at the tube walls and decreased intensity towards the center. In addition to BiI_3_ nanotubes, we observed BiI_3_ nanorods within CNT. The STEM-EDX elemental maps on the BiI_3_ nanorod@CNT (BiI_3_ NR@CNT) show the distribution of Bi and I and the quantification of the EDX spectra established the 1:3 ratio of Bi:I as in BiI_3_ (Supplementary Fig. S2). EELS measurement on the encapsulated nanorods show Bi M_4, 5_ and I M_4, 5_ edges, with edge onsets at 2604 eV and 628 eV, respectively, confirming the encapsulation of BiI_3_ (Supplementary Fig. S2). Supplementary Figs S3 and S4 show the crystal orientation of BiI_3_ nanorods in two different variants; [100] and [−111] of rhombohedral BiI_3_.

**Figure 4:**
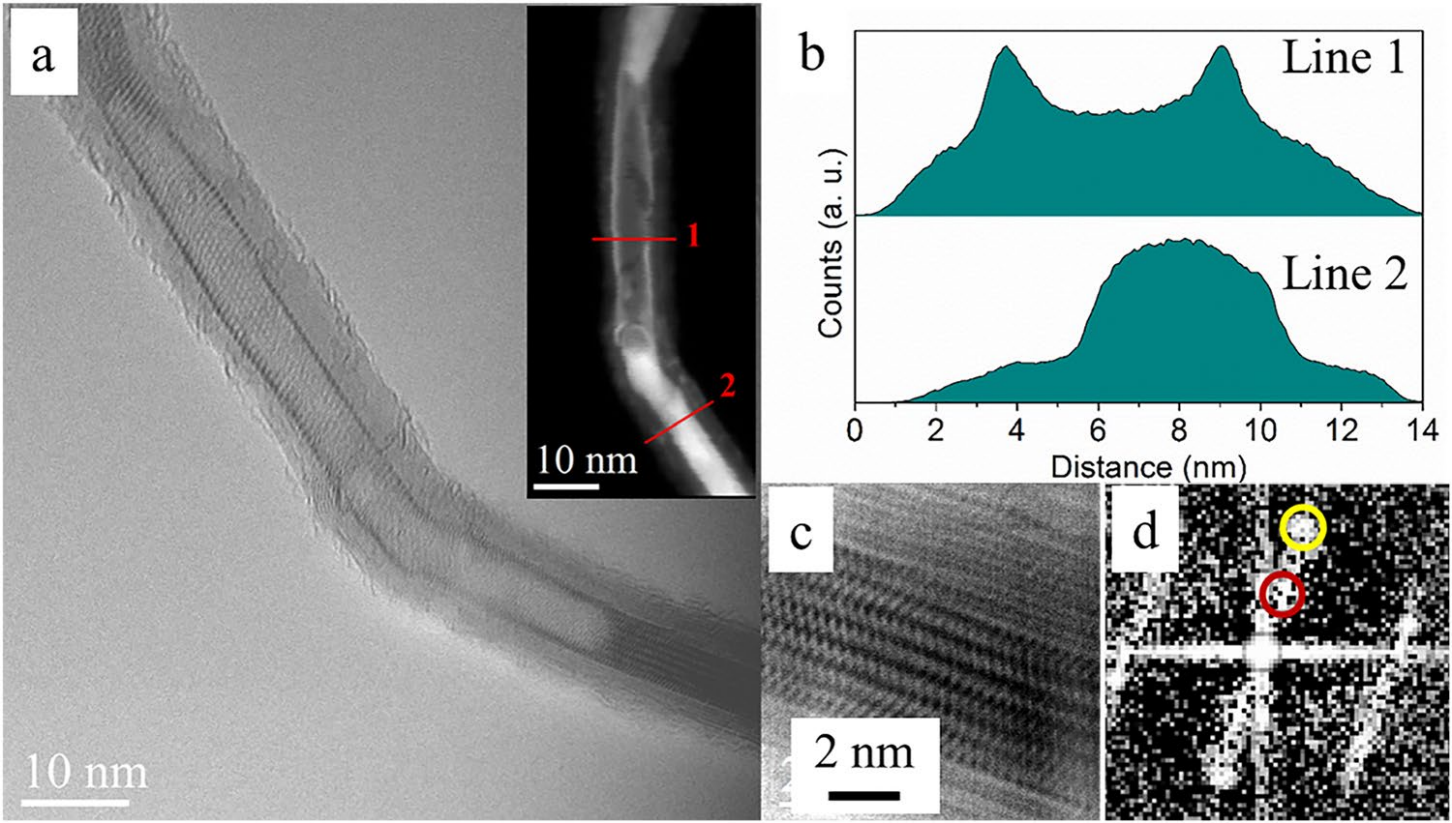
Partially filled BiI_3_ NT@CNT. (**a**) HRTEM image (HAADF-STEM image as inset). (**b**) The intensity profile along line 1 and 2 in the inset showing the hollow BiI_3_ NT@CNT and the filled BiI_3_ NT@CNT. (**c**) An enlarged image of the filled region in a. (**d**) Corresponding FFT indicating the orientation relationship between BiI_3_ (003) (red circle) and CNT (002) (yellow circle).

In addition to single walled nanotubes and nanorods of BiI_3_, there were nanotubes which appear to be partially filled. Figure 4a shows a BiI_3_@CNT where a single walled BiI_3_ nanotube is observed within MWCNT as well as regions where the BiI_3_ NT is filled resulting in a rod-like appearance. Figure 4b shows the HAADF intensity profiles across the tube at position 1 and 2 shown in inset Figure 4a. The analysis confirms that the BiI_3_ NT is present as single walled and filled, at positions 1 and 2, respectively. The (003) planes visible in the filled region shown in Fig. 4c, show an epitaxial relation with the (002) planes of the MWCNT template (Fig. 4d).

**Figure 5:**
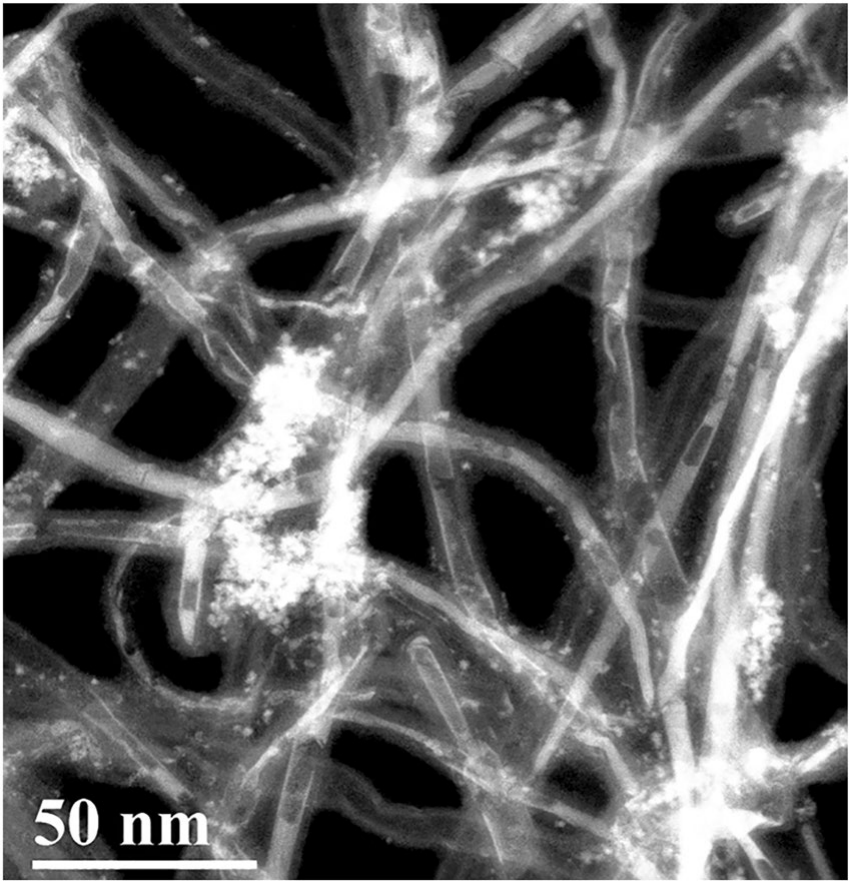
Low magnification image of BiI_3_@CNT showing encapsulation as nanotubes, partially filled nanotubes and nanorods.

Low magnification images showing the encapsulation of BiI_3_ as nanorods, nanotubes and partially filled nanotubes are shown in Fig. 5 and Supplementary Fig. S5. From the electron microscopy images, the yield of encapsulation is observed to be greater than 80%. In order to identify the MWCNT diameter regime where the formation of single walled BiI_3_ nanotubes is favorable, we performed statistical analysis of the samples obtained from the host MWCNTs of different diameters. Supplementary Fig. S6 shows the inner diameter distribution of four MWCNT samples investigated. CNT 1, with a mean inner diameter of 2.31 nm resulted in exclusive formation of nanorods. CNTs with larger diameters (CNT 2, CNT 3 and CNT 4) facilitated the formation of single walled BiI_3_ nanotubes along with nanorods of BiI_3_. Supplementary Fig. S7 shows the frequency distribution of BiI_3_ nanotubes and nanorods depending on the MWCNT diameters. A preferential formation of nanotubes at larger diameters is observed in all the latter three CNT samples. In a previous report on PbI_2_@CNT, a range of diameters between 4 to 8 nm was suggested to facilitate the formation of nanotubes^3^. Herein, a host inner diameter of ~3 nm is identified as the lowest threshold for the formation of single-walled BiI_3_ nanotubes and nanotubes as large as 12 nm in diameter were also observed in the CNTs. In addition, the possibility of formation of NTs and NRs of other halides of bismuth is elucidated with the example of BiCl_3_ (Supplementary Figs S8–S10).

In order to understand the relative stability and possible modulation of electronic properties of BiI_3_ in the nanotubular state, DFT calculations have been employed. Preliminary geometry optimizations of both the bulk BiI_3_ and a free-standing single sheet BiI_3_ do not show evidence about a distortion of layers’ hexagonal symmetry. Isolated BiI_3_ layer undergoes a minor in-plane contraction, adopting the equilibrium lattice parameter a = 7.26 Å (for the bulk a = 7.52 Å).

**Figure 6:**
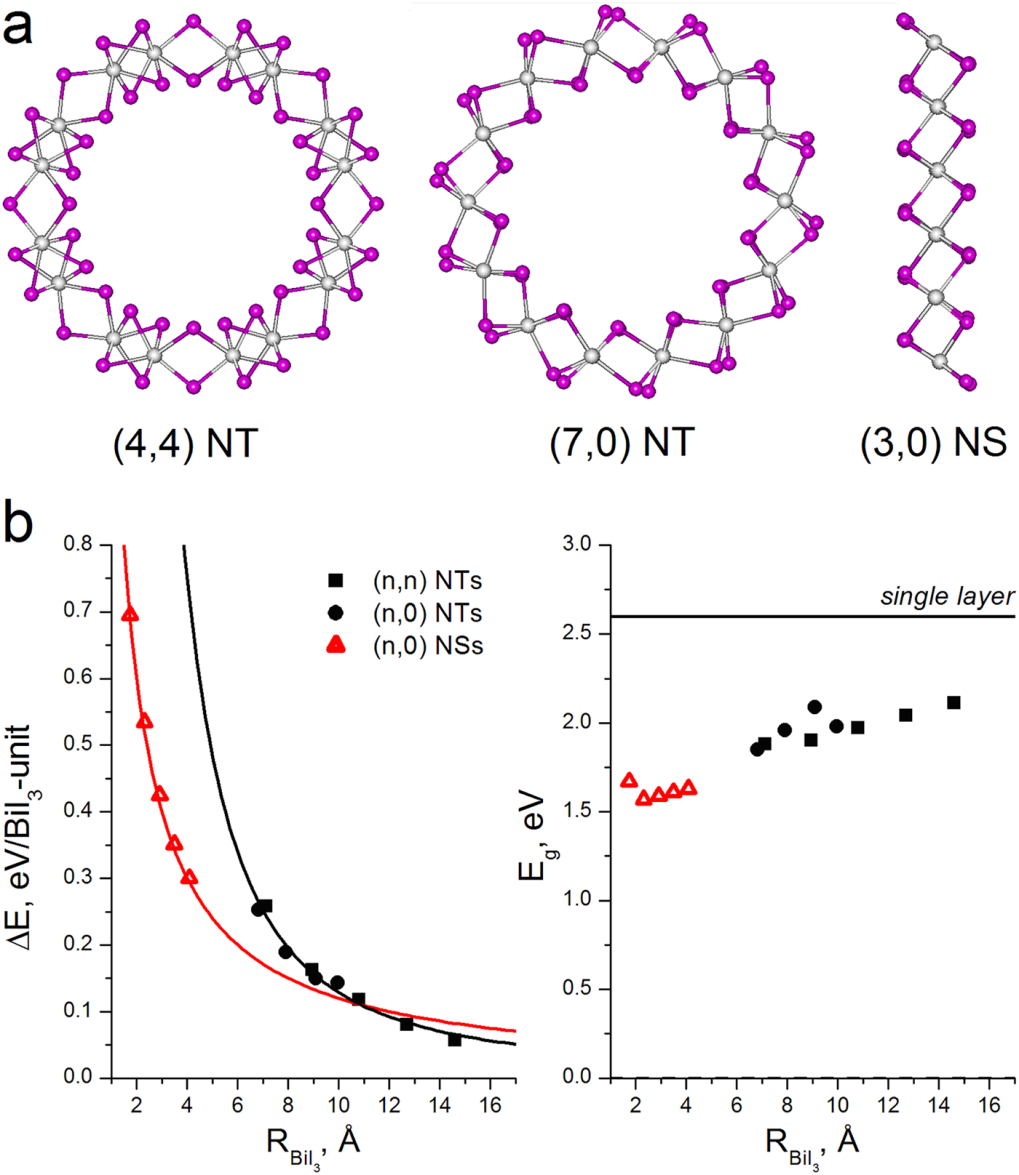
(**a**) Ball-and-stick models of exemplary one-dimensional single-walled BiI_3_ nanostructures after geometry optimization using DFT method: *armchair* (4,4) and *zigzag* (7,0) nanotubes, *zigzag* (3,0) nanostripe (the views along main axes, Bi and I atoms are in white and violet). (**b**) Strain energies ΔE and band gaps E_g_ for different BiI_3_ nanostructures depending on their radii (for a nanostripe the radius corresponds to the width normalized by 2π).

The construction principles of BiI_3_ SWNTs coincide with those for the well-known graphene-derived CNTs. The TEM images of BiI_3_ SWNTs encapsulated into MWCNTs suggest that they are likely chiral and may possess even a variable chirality along the axis of bent MWCNTs. Hence, the nanotubes of two extreme chiralities have been considered: armchair (n,n) with n = 3–7 and zigzag (n,0) with n = 5–8 (Fig. 6a), which are computationally also less demanding, than chiral nanotubes. The properties of nanotubes were compared to those of BiI_3_ nanostripes - as single layer nanorods - with both glide- and mirror-symmetric armchair edges: zigzag (n,0) with n = 2, 3 and chiral (n,1) with n = 1–3 (Fig. 6a). To decrease computational demands, our study of nanorods was limited only to the single-layer members. Yet, the main contribution into excessive total energy of both nanorods and nanostripes arises from the energy of dangling bonds at the edges, and a weak interlayer (mainly van der Waals) interaction may be neglected here.

Preservation of morphological integrity after geometry optimization has been observed for all BiI_3_ nanostructures irrespective of the chirality type in all range of chosen indices. To trace the relative stability as a function of both the radius and the chirality of nanotubes and nanostripes, their excessive energies ΔE were defined as the energies relative to the energy of the BiI_3_ monolayer. Hence, the main contribution to ΔE, for nanotubes or flat nanostripes, is due to the pure strain energy and the energy of dangling bonds at edges, respectively. The values of ΔE per BiI_3_-unit are collected in Fig. 6b. The data confirm that, the main factor defining the morphological stability of cylindrical nanotubes is the radius R. Irrespective of the chirality, the DFT calculated values ΔE for SWNTs can be interpolated using a single function, obeying the ~1/R^2^ rule predicted in the framework of classical elasticity theory for a bent layer.

**Figure 7:**
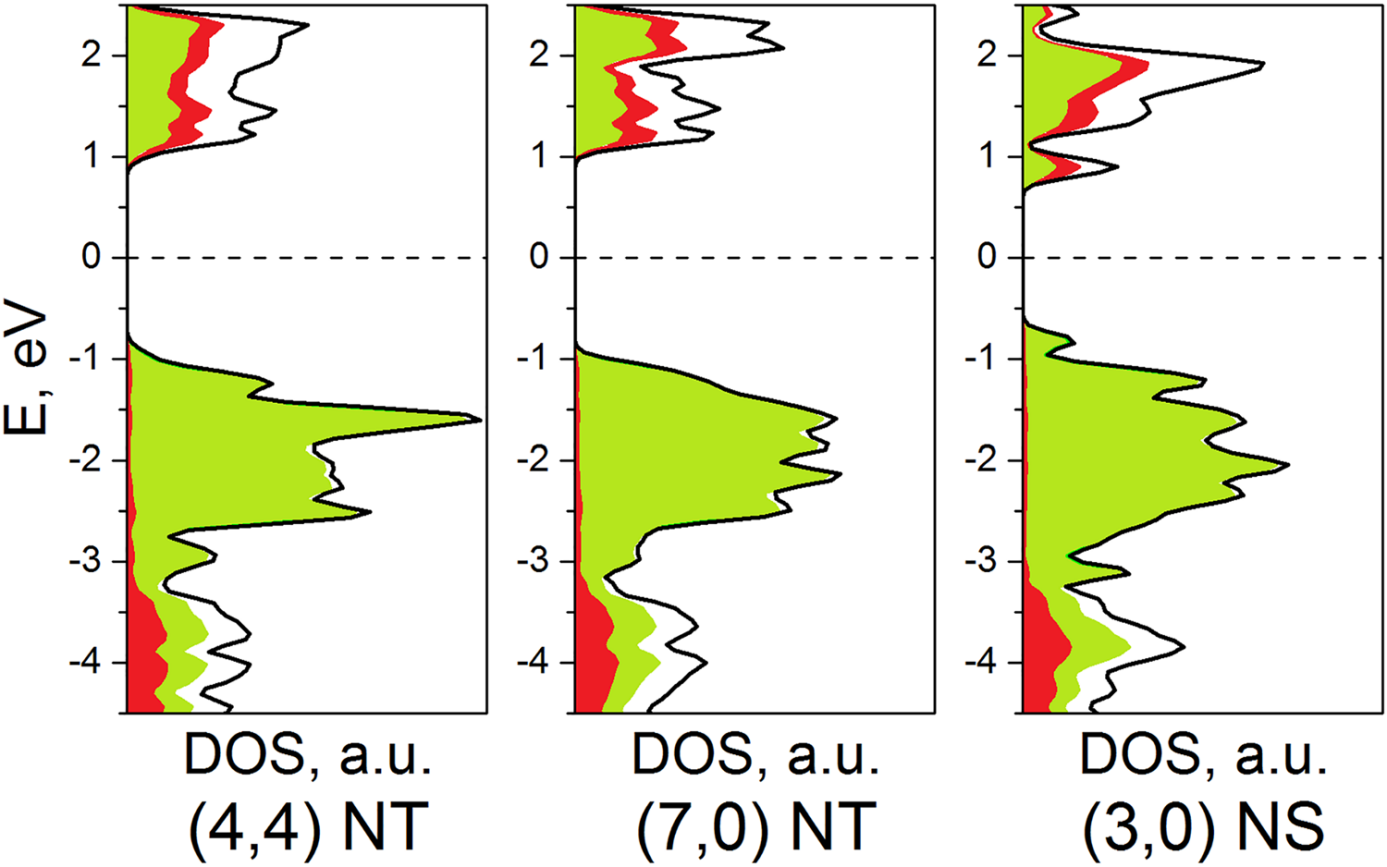
Densities-of-states (DOS) for exemplary one-dimensional BiI_3_ nanostructures (Fermi level is set to 0.0 eV). Total DOS is outlined in black, valence Bi6*p-* and I5*p*-states are painted in red and green, respectively.

The proportionality factor between ΔE and 1/R^2^ for all considered BiI_3_ nanotubes is found to be equal to 11.87 (eV·Å^2^)/unit or 3.0 (eV·Å^2^)/atom. This factor depends essentially on the layers’ thickness and lies between the previously reported values of 0.5 (eV·Å^2^)/atom and 16.1 (eV·Å^2^)/atom, for SWCNT and MoS_2_ nanotubes, respectively^29^. Noteworthy, that both carbon and MoS_2_ nanotubes can be produced in large amounts nowadays. Therefore, our calculations reveal, the elastic strain of the BiI_3_ layers should not be the main constraint to a future mass synthesis of the BiI_3_ nanotubes. The lower boundary of the stability of BiI_3_ SWNTs is predestined by the energy of dangling bonds at the edges of corresponding BiI_3_ nanostripes as unrolled nanotubes. The values of ΔE per BiI_3_-unit for nanostripes obey ~1/R rule (Fig. 6b), which reflects a negligible dependence of the energy of dangling bonds on the width and an identical geometry reconstruction of the edges at different nanostripes. However, the most important outcome can be found in the cross-point of ΔE functions for nanostripes and nanotubes. BiI_3_ SWNTs become thermodynamically more stable at diameters larger, than 2.2 nm. The calculated band structures and the densities of electronic states (DOS) for selected nanotubes and nanostripes of BiI_3_ are visualized in Fig. 7 and Supplementary Fig. S11. The DOS profile and relative location of the bands observed for different single walled nanostructures can be found qualitatively similar, exhibiting also a very close similarity with those of the single layer. In accordance to the polar covalent character of the Bi-I bonding, the valence band at −1…−3 eV is composed of mainly I5p-states, while the valence band at −3…−5 eV and the bottom of conduction band are the mixtures of I5p- and Bi6p-states.

All the studied BiI_3_ nanostructures are semiconductors likely with the direct band gaps at Г-point, yet, with too narrow dispersions at the occupied and conduction edges of the band gaps. The band gap values do not fall below 1.5 eV and in the case of SWNTs they approach the value for the single BiI_3_ layer 2.5 eV (Fig. 7, Supplementary Fig. S11). The latter value is found overestimated in agreement with previous theoretical study^30^, since no spin-orbit coupling was accounted in our calculations of nanostructures. While the fundamental band gap of BiI_3_ remains disputable, our calculations unveil a tendency in modulation of the band gap of enrolled BiI_3_ layers^31^, hence, the nanotubes, too. The vanishing of their fundamental band gaps at low R can be explained by a strengthened overlapping between the Bi6p- and I5p-orbitals at interior side of nanotube, yet, not manifesting itself through the localized states in the fundamental band gap, which is typical for the dangling bonds. The band gap modulation for flat BiI_3_ nanostripes has another background. In fact, their band structure demonstrates the same fundamental band gap ~2.5 eV as that for the single layer. Yet, several states split off from both the conduction and the valence bands, leading to formal decrease of the band gap. These localized states are associated with the edge atoms of a low coordination: five-fold Bi and monodentate I atoms, respectively.

Regrettably, direct DFT calculation with correction on spin-orbit coupling is computationally too expensive for BiI_3_ nanostructures due to the geometric and atomistic sizes of their unit cells. However, our preliminary estimations of the band gaps should contain only a systematic error and should qualitatively reproduce the trends in band gap modulations of different nanotubes and nanostripes. The folding of a planar layer into cylindrical nanotube facilitates the orbitals’ overlapping at internal side of nanotube. Noteworthy, this situation is equivalent to the external pressure applied to the bulk BiI_3_. Devidas *et al*. have demonstrated that at a high pressure the band gap of the bulk polymorphs of BiI_3_ gets decreased in experiment as well as in both schemes of DFT calculations: without and with spin-orbit coupling^32^.

## Conclusions

In conclusion, capillary filling was employed for obtaining bismuth halides encapsulated within MWCNTs. In the case of BiI_3_, we have observed single walled nanotube with hexagonal crystal structure within the MWCNT host. Such core-shell nanotubes could lead to novel fundamental properties and also applications. Diameter dependence of the nanotube formation versus nanorods were analyzed using statistics obtained from electron microscopy images. Our results show a lower threshold inner diameter of the host MWCNT of ~3 nm, which is the minimum diameter for the formation of BiI_3_ single-walled nanotubes. These experimental observations agree very well with the DFT calculations which have been carried out in the present study to understand the energetics of their formation.

## Methods

### Synthesis and Characterization

The encapsulation of BiI_3_ and BiCl_3_ in MWCNT was carried out by capillary filling. The bismuth halide along with CNT, vacuum sealed in a quartz ampule, was annealed in a furnace above the melting point of the halide and subsequently cooled to room temperature. The conditions for the synthesis of BiI_3_@CNT and BiCl_3_@CNT are given in Tables S1 and S2, respectively. Samples for TEM/STEM analysis were prepared on holey carbon film 300 mesh Cu grids. A FEI Titan Themis 60–300 kV electron microscope, operated at 80 kV, equipped with probe and image correctors and a monochromator, was used for TEM and STEM imaging. EDX spectra were obtained using a Super-X detector and EELS measurements were carried out on an Enfinium ER spectrometer. Crystal models were built using Crystal maker software and diffraction patterns were simulated using the Single crystal software.

### Computational Details

The DFT calculations within the Generalized Gradient Approximation after Perdew-Burke-Ernzerhof parametrization were performed using the SIESTA 4.0 package^33^. The norm-conserving Troullier–Martins pseudopotentials were used for the description of the core electrons^34^, while the valence electron shells were adapted as 5*s*^2^5*p*^5^ for I and 6*s*^2^6*p*^3^ for Bi with the pseudopotential core radii corresponding to 2.24 a_B_ and 2.26 a_B_ for I5*s*- and I5*p*-states, 2.88 a_B_ for all Bi states. The valence orbitals were described using double zeta basis set, including polarization functions. The numerical integrations were performed using the real-space grid with the 300 Ry energy cutoff. The *k*-point mesh for periodic systems established after Monkhorst - Pack and a cutoff of 15 Å were employed for *k*-point sampling^35^. For 1D and 2D structures the lattice parameter in “non-periodic” directions was chosen as 40 Å. Both the lattice parameters and the atomic positions of a unit cell were optimized until the total residual force and a residual stress component have achieved 0.05 eV/Å and 0.1 GPa, respectively.

### Data availability

All data generated or analysed during this study are included in this published article (and its Supplementary Information files).

## Electronic supplementary material

Supplementary Information
