# Supplementary material for: Single Walled BiI3 Nanotubes Encapsulated within Carbon Nanotubes
Source: Sci Rep. 2018 Jul 4;8:10133. doi: 10.1038/s41598-018-28446-2 (PMC6031677; doi:10.1038/s41598-018-28446-2)

Supplementary information

Single Walled BiI_3 Nanotubes Encapsulated within Carbon Nanotubes

Anumol Erumpukuthickal Ashokkumar,[1] Andrey N. Enyashin[2, 3] and Francis Leonard Deepak[1]

[1] Nanostructured Materials Group, Advanced Electron Microscopy, Imaging and Spectroscopy, International Iberian Nanotechnology Laboratory (INL), Av. Mestre José Veiga s/n, 4715-330 Braga - Portugal

[2] Institute of Natural Sciences and Mathematics, Ural Federal University, Turgeneva Str., 4, 620083 Ekaterinburg, Russian Federation

[3]Institute of Solid State Chemistry, Ural Branch of Russian Academy of Sciences, Pervomayskaya Str., 91, 620990 Ekaterinburg, Russian Federation

Table of Contents

1. Experimental Procedures

Table S1**.** Synthesis conditions for BiI_3@CNT.

Table S2. Synthesis conditions for $BiCl_3$@CNT

2. Results and Discussion

2a. BiI_3 Nanotube@CNT

Figure S1. Structural analysis of the BiI_3 NT@CNT. a) AC-TEM image of a BiI_3 crystal inside CNT, b) Corresponding FFT which shows that the crystal orientation is [001]. c) Structural model of BiI_3 NT in [001] orientation and d) Simulated diffraction pattern of the [001] zone axis of rhombohedral BiI_3 which matches with the FFT pattern in b.

Table S3**.** EDX quantification of BiI_3 NT@CNT.

2b. BiI_3 Nanorod@CNT

Figure S2**.** BiI_3 NR@CNT 1. a) HAADF-STEM image and EDX elemental maps showing C, Bi and I. b) EDX spectrum showing C, Bi, I peaks. c and d: EELS spectra showing the I $M_{4,5}$ edge and Bi $M_{4,5}$ edge.

Figure S3. Structural analysis of the BiI_3 NR@CNT. a) AC-TEM image of a BiI_3 crystal inside CNT, b) Corresponding FFT which shows that the crystal orientation is [100]. c) Structural model of BiI_3 in [100] orientation and d) Simulated diffraction pattern of the [100] zone axis of rhombohedral BiI_3 which matches with the FFT pattern in b.

Figure S4**.** Structural analysis of the BiI_3 NR@CNT. a) HRTEM image of a BiI_3 crystal inside CNT, b) Corresponding FFT which shows that the crystal orientation is [-111]. Inset a, shows the corresponding HAADF-STEM image. c) Structural model of BiI_3 in [-111] orientation and d) Simulated diffraction pattern of the [-111] zone axis of rhombohedral BiI_3 which matches with the FFT pattern in b.

Figure S5**.** Low magnification HAADF-STEM images showing the encapsulation of BiI_3 as rods and tubes within MWCNT.

2c. Influence of CNT diameter on Rod versus Tube morphology of BiI_3

Figure S6**.** Diameter distribution of the carbon nanotube samples; CNT 1 (a), CNT 2 (b), CNT 3 (c), CNT 4 (d)

Figure S7. Diameter distribution of the BiI_3 nanotube and nanorod formed inside various CNT samples. CNT 2 (a), CNT 3 (b), CNT 4 (c)

2d. $BiCl_3$@CNT

Figure S8. HAADF-STEM image of $BiCl_3$@CNT showing nanotube and nanorod morphology of $BiCl_3$ encapsulation in CNT.

Figure S9**.** $BiCl_3$ NR@CNT. a) HAADF-STEM image and the EDX elemental maps showing C, Bi and I. b) Corresponding EDX spectrum.

Figure S10**.** Structural analysis of the $BiCl_3$ NR@CNT. a) HAADF-STEM image and b) AC-TEM image of a $BiCl_3$ crystal inside CNT, c) Corresponding FFT which shows that the crystal orientation is [001]. d) Simulated diffraction pattern of the [001] zone axis of orthorhombic $BiCl_3$ which matches with the FFT pattern in c.

2e. DFT Calculation

Figure S11. Band structure and densities-of-states (DOS) for exemplary one-dimensional BiI_3 nanostructures (Fermi level is set to 0.0 eV). Total DOS is depicted as full black line, valent Bi6*p*- and I5*p*-states are painted in red and green, respectively. DFT calculations without spin-orbit correction.

1. **Experimental Procedures**

Table S1. Synthesis conditions for BiI_3@CNT.

Sample	CNT:BiI_3 (mg)	Temperature (°C)	Heating rate (°C/min)	Duration (h)
BiI_3 CNT 1	11:200	500	2	72
BiI_3 CNT 2	10:200	500	2	72
BiI_3 CNT 3	10:200	500	2	72
BiI_3 CNT 4	10:200	500	2	72

Table S2. Synthesis conditions for $BiCl_3$@CNT

Sample	CNT:$BiCl_3$ (mg)	Temperature (°C)	Heating Rate (°C/min)	Duration (h)
$BiCl_3$ CNT	10:300	350	2	72

2. Results and Discussion

2a. BiI_3 Nanotube@CNT

Figure S1. Structural analysis of the BiI_3 NT@CNT. a) AC-TEM image of a BiI_3 crystal inside CNT, b) Corresponding FFT which shows that the crystal orientation is [001]. c) Structural model of BiI_3 NT in [001] orientation and d) Simulated diffraction pattern of the [001] zone axis of rhombohedral BiI_3 which matches with the FFT pattern in b.

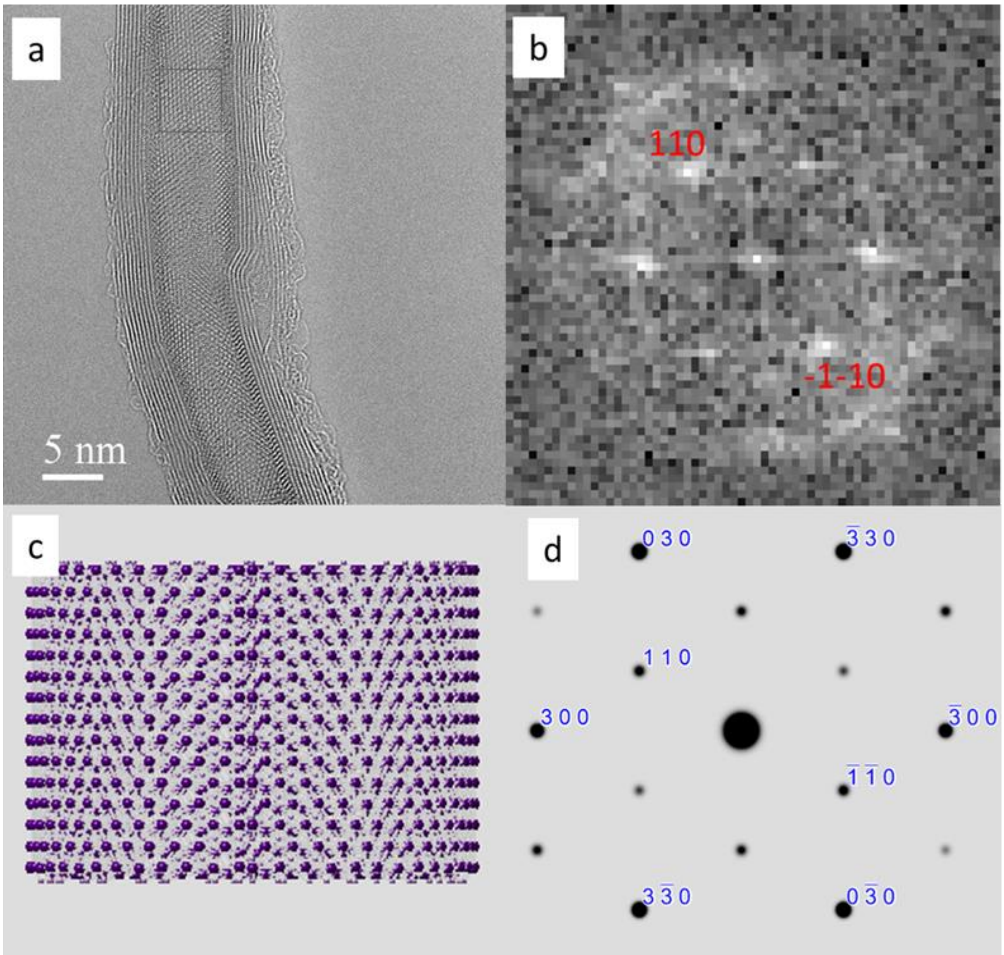

Table S3. EDX quantification of BiI_3 NT@CNT.

Element	Series	norm. C [wt.%]	Atom. C [at.%]
Bismuth	M-series	35.09	24.72
Iodine	L-series	64.91	75.28
	Total:	100.00	100.00

2b. BiI_3 Nanorod@CNT

Figure S2. BiI_3 NR@CNT 1. a) HAADF-STEM image and EDX elemental maps showing C, Bi and I. b) EDX spectrum showing C, Bi, I peaks. c) and d) show the EELS spectra of the I $M_{4,5}$ edge and Bi $M_{4,5}$ edge.

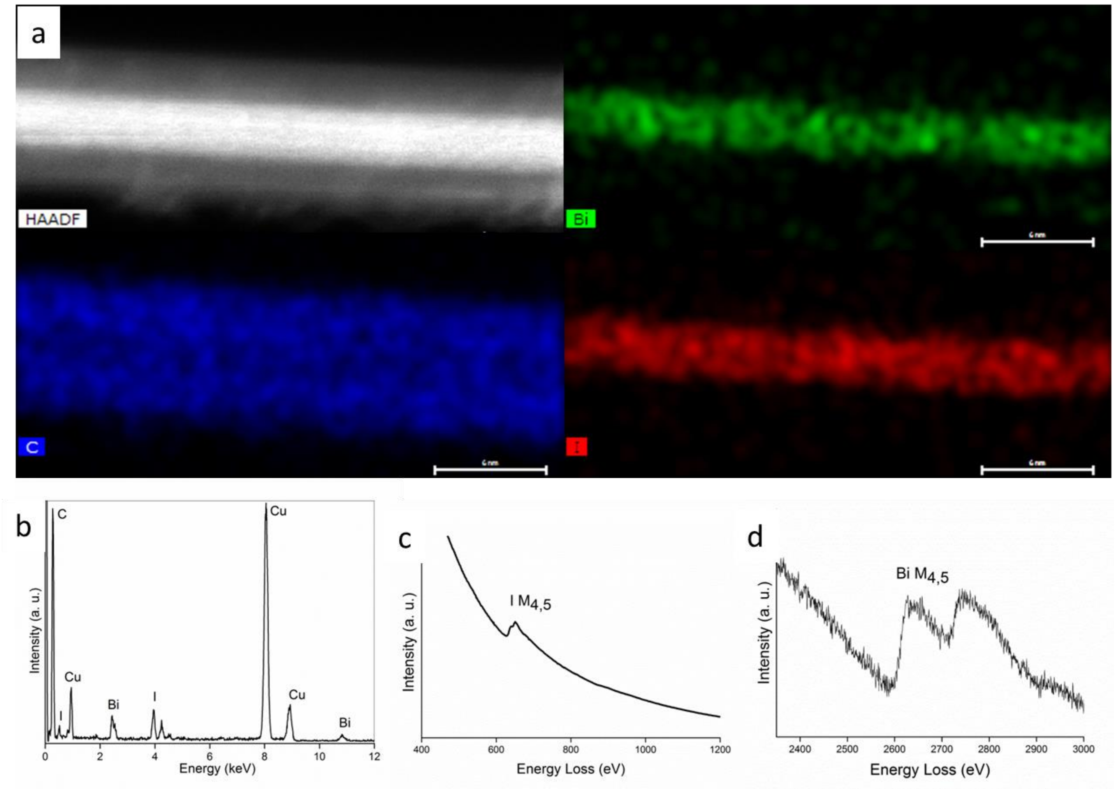

Figure S3. Structural analysis of the BiI_3 NR@CNT. a) AC-TEM image of a BiI_3 crystal inside CNT, b) Corresponding FFT which shows that the crystal orientation is [100]. c) Structural model of BiI_3 in [100] orientation and d) Simulated diffraction pattern of the [100] zone axis of rhombohedral BiI_3 which matches with the FFT pattern in b.

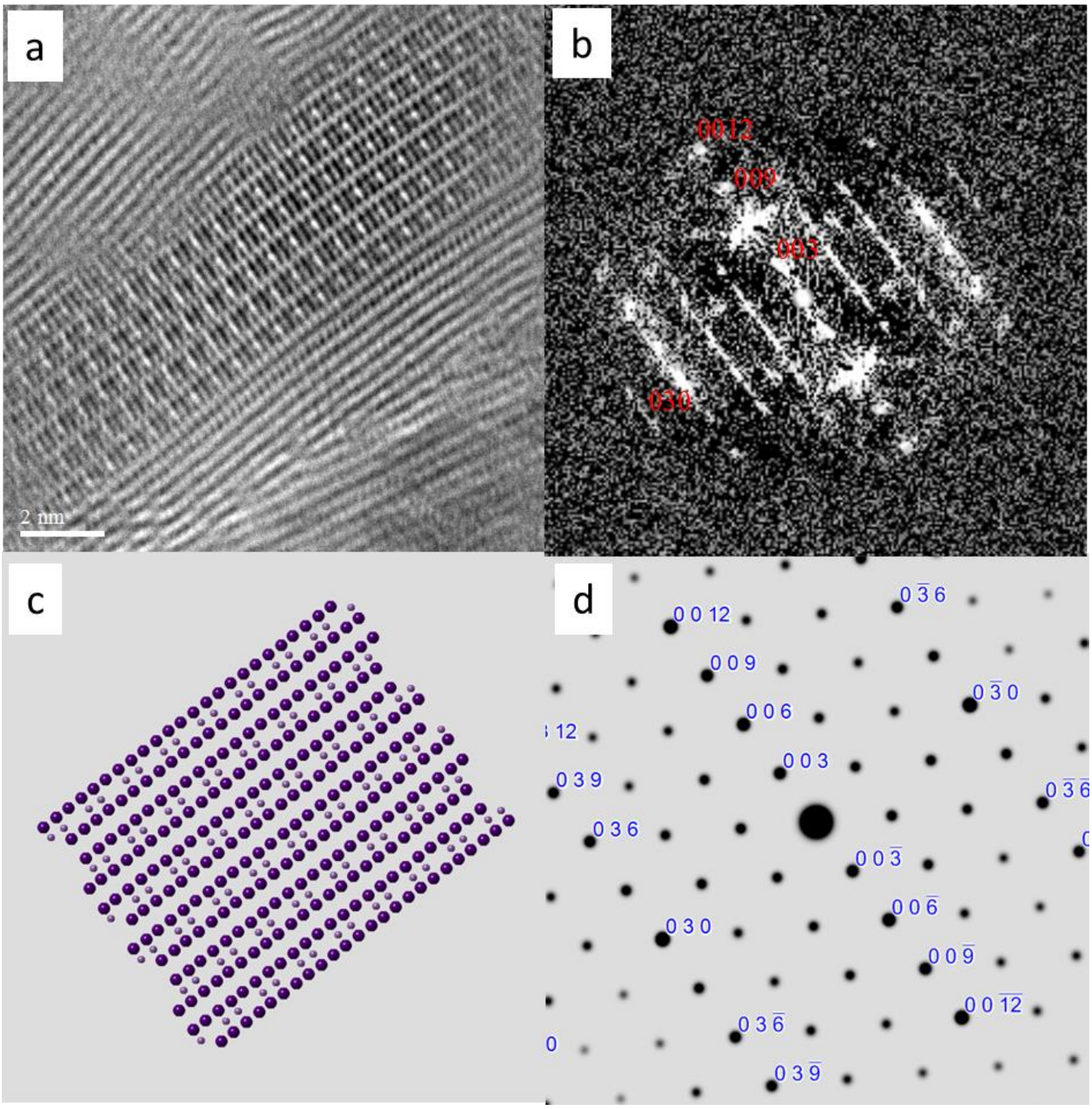

Figure S4. Structural analysis of the BiI_3 NR@CNT. a) HRTEM image of a BiI_3 crystal inside CNT, b) corresponding FFT which shows that the crystal orientation is [-111]. Inset a, shows the corresponding HAADF-STEM image. c) Structural model of BiI_3 in [-111] orientation and d) Simulated diffraction pattern of the [-111] zone axis of rhombohedral BiI_3 which matches with the FFT pattern in b.

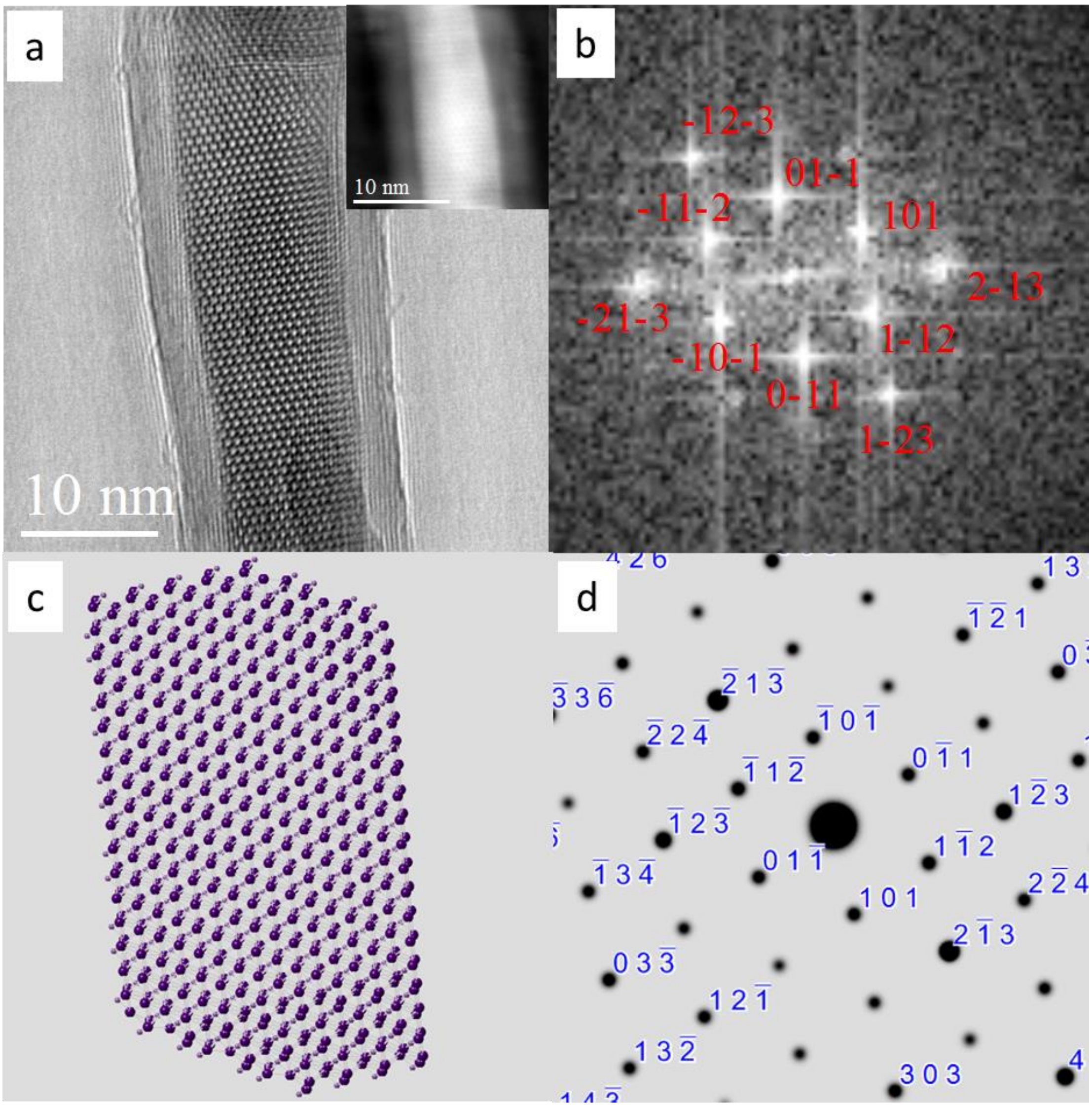

Figure S5. Low magnification HAADF-STEM images showing the encapsulation of BiI_3 as rods and tubes within MWCNT.

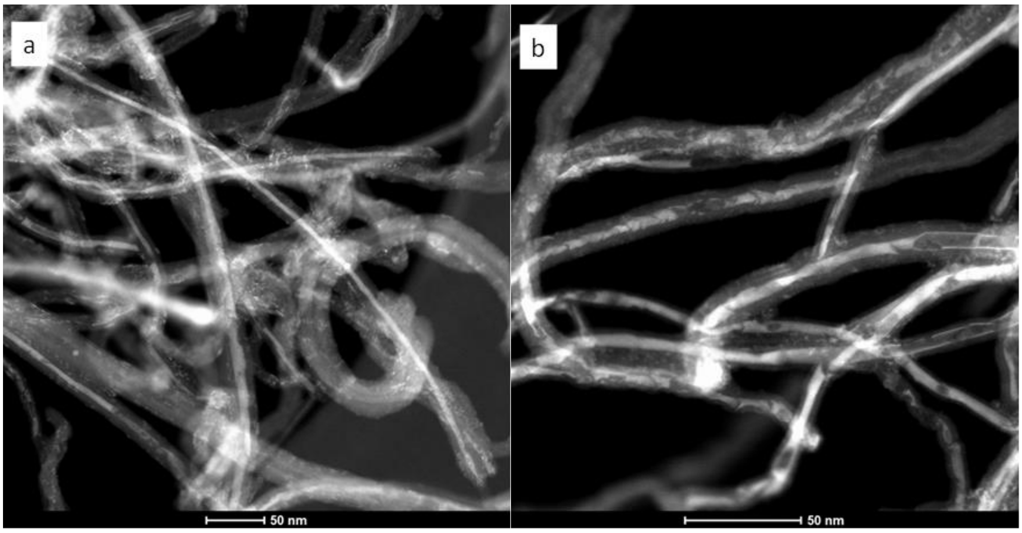

2c. Influence of CNT diameter on rod versus tube morphology of BiI_3

Figure S6. Diameter distribution of the carbon nanotube samples; CNT 1 (a), CNT 2 (b), CNT 3 (c), CNT 4 (d)

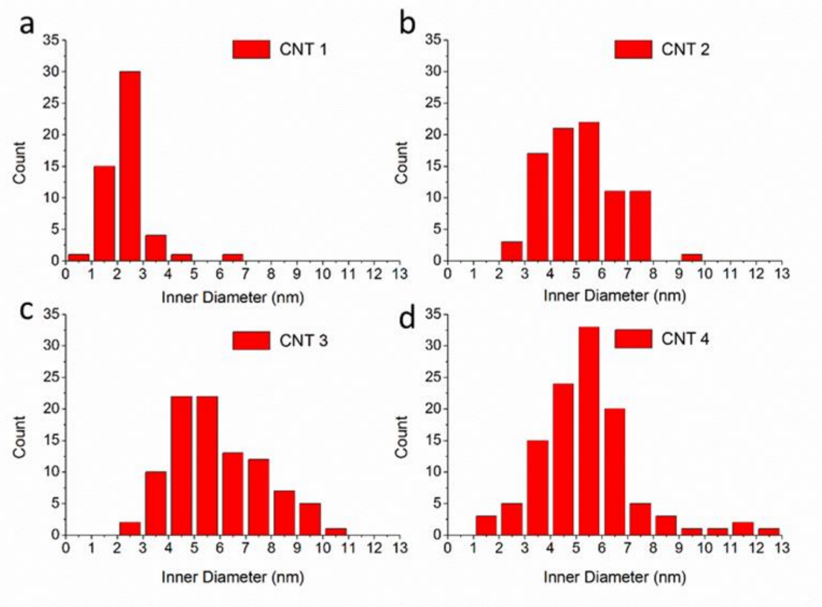

Figure S7. Diameter distribution of the BiI_3 tube and rod formed inside various CNT samples. CNT 2 (a), CNT 3 (b), CNT 4 (c)

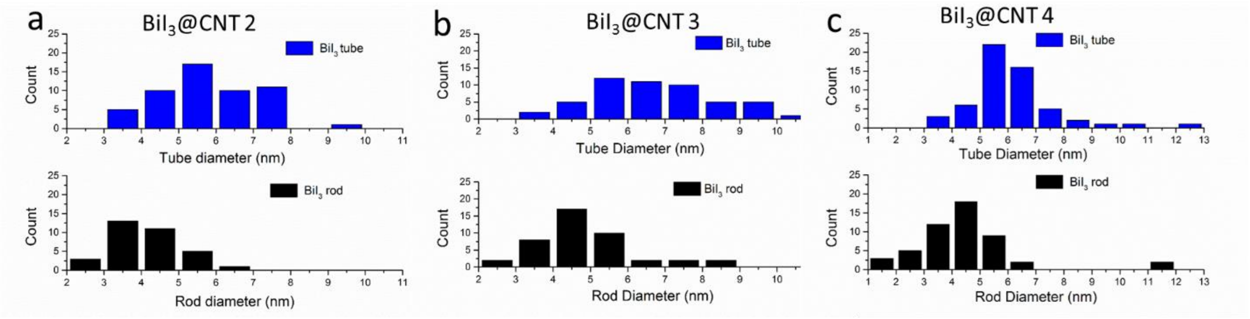

2d. $BiCl_3$@CNT

Extending the study to other bismuth halides, by annealing $BiCl_3$ along with MWCNT at 350°C in vacuum sealed quartz ampules, $BiCl_3$ was encapsulated within the MWCNTs. Figure S8 shows the low magnification image of the $BiCl_3$@CNT with tube-like and rod-like $BiCl_3$ in CNT. Figure S9 shows the distribution of Bi and Cl within the CNT and also the corresponding EDX spectrum which on quantification gives Bi:Cl ratio consistent with that of $BiCl_3$. The filling was predominantly amorphous in nature although crystalline $BiCl_3$ was observed. The analysis of HRTEM image of the crystalline filling indicates the presence of orthorhombic $BiCl_3$ crystal (ICSD PDF Number: 00-024-1003) within CNT (Figure S10).

Figure S8. HAADF-STEM image of $BiCl_3$@CNT showing nanotube and nanorod morphology of $BiCl_3$ encapsulation in CNT.

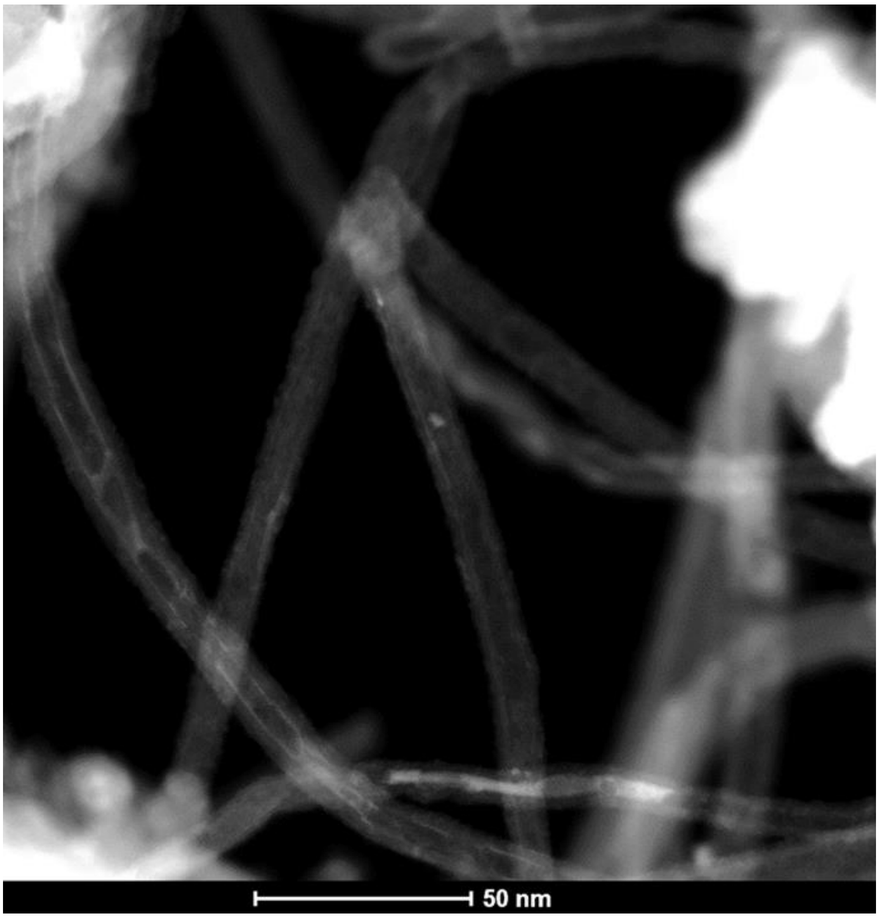

Figure S9. $BiCl_3$ NR@CNT. a) HAADF-STEM image and the EDX elemental maps showing C, Bi and I. b) Corresponding EDX spectrum.

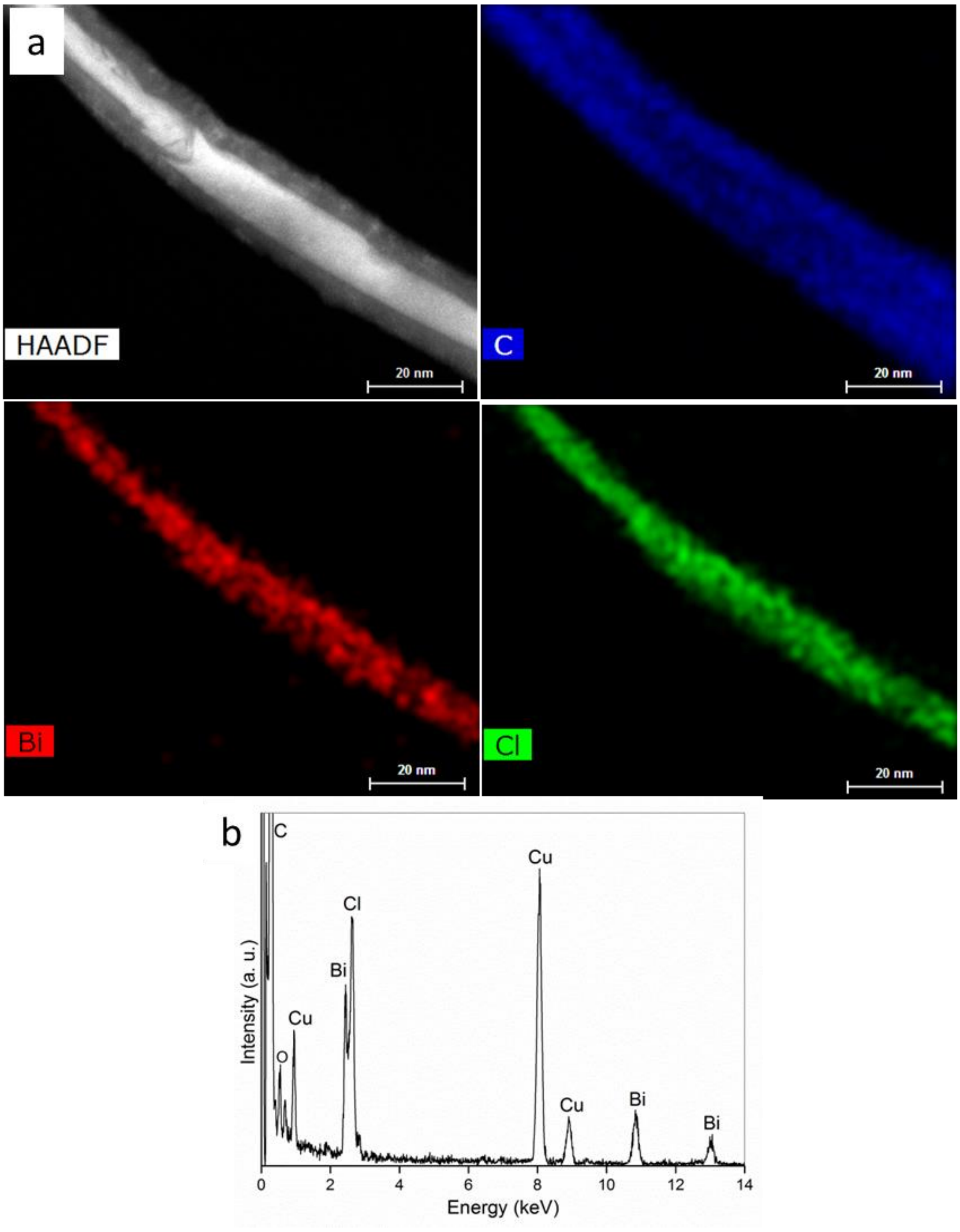

Figure S10. Structural analysis of the $BiCl_3$ NR@CNT. a) HAADF-STEM image and b) AC-TEM image of a $BiCl_3$ crystal inside CNT, c) Corresponding FFT which shows that the crystal orientation is [001]. d) Simulated diffraction pattern of the [001] zone axis of orthorhombic $BiCl_3$ which matches with the FFT pattern in c.

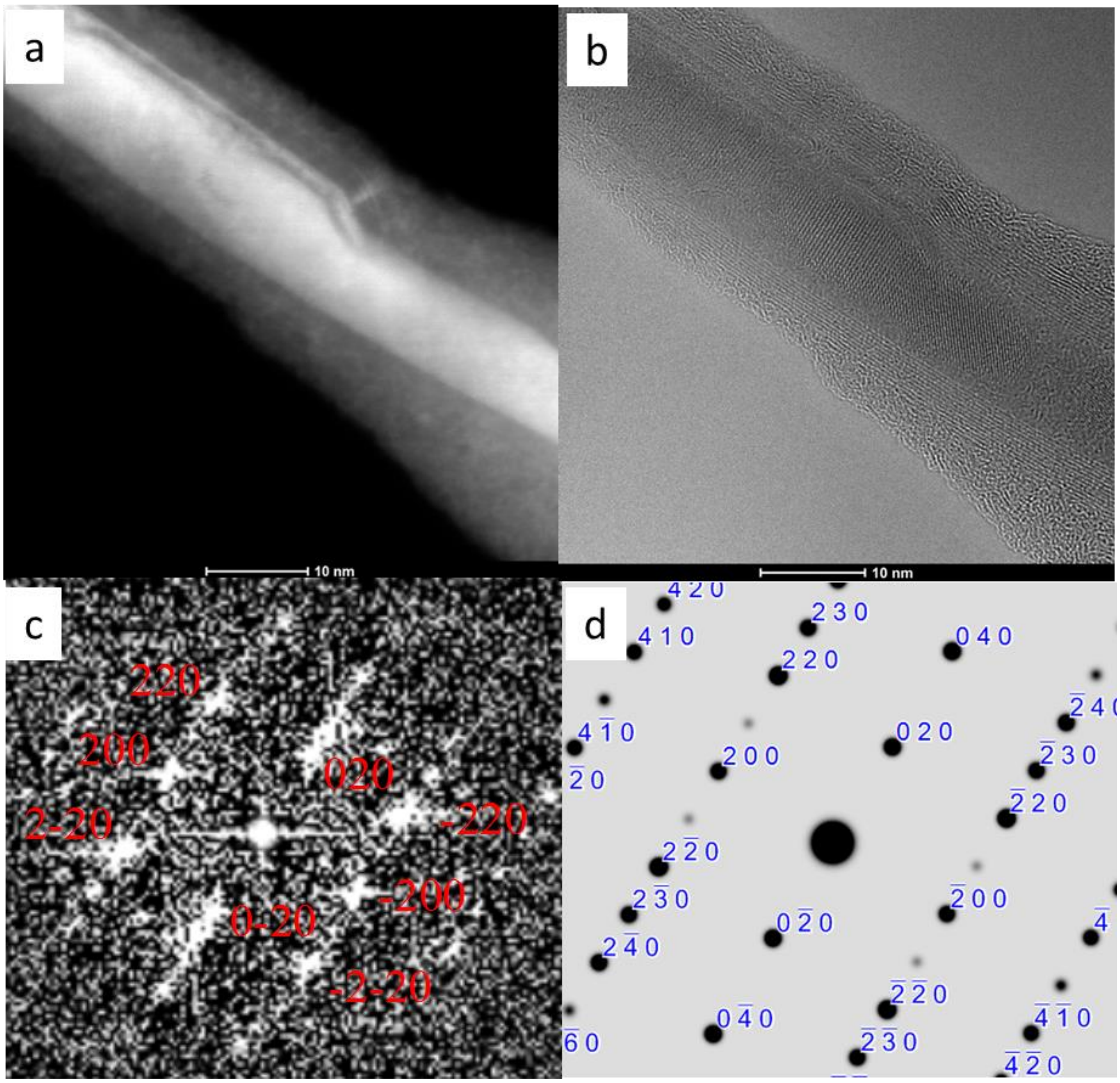

2e. DFT Calculation

Figure S11. Band structure and densities-of-states (DOS) for exemplary one-dimensional BiI_3 nanostructures (Fermi level is set to 0.0 eV). Total DOS is depicted as full black line, valent Bi6*p*- and I5*p*-states are painted in red and green, respectively. DFT calculations without spin-orbit correction.

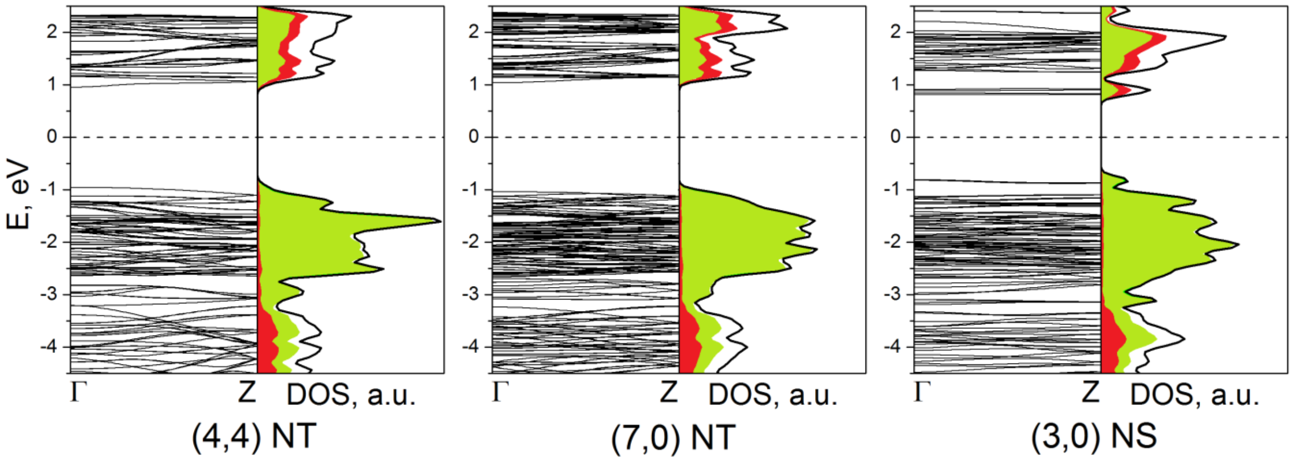

Supplement: Supplementary file 1 — Supplementary Information [file 41598_2018_28446_MOESM1_ESM.pdf]
